# Screening for insomnia disorders among flight crews in Tunisia

**DOI:** 10.1192/j.eurpsy.2025.902

**Published:** 2025-08-26

**Authors:** A. Chouchane, I. Jemmeli, A. Gaddour, M. Bouhoula, I. Kacem, A. Aloui, M. Maoua, A. Brahem, H. Kalboussi, O. El Maalel, S. Chatti, N. Mrizak

**Affiliations:** 1 Occupational Medicine and Professional Pathologies Department, Farhat Hached Teaching Hospital, University of Sousse, Faculty of Medicine of Sousse, Sousse, Tunisia; 2 Occupational Medicine Department- Ibn El Jazzar Hospital, Kairouan, 2- University of Sousse, Faculty of Medicine of Sousse, Sousse, Tunisia; 3 Occupational Medicine Department- Ibn El Jazzar Hospital, Kairouan, University of Sousse, Faculty of Medicine of Sousse, Sousse, Tunisia, Sousse, Tunisia

## Abstract

**Introduction:**

Flight crew members are subjected to various stressors that can disrupt their sleep-wake cycle, leading to a higher prevalence of sleep disorders.

**Objectives:**

to assess the prevalence of sleep disturbances among flight crew members of a private airline company in Tunisia.

**Methods:**

A cross-sectional study was conducted involving flight crew members employed by a private airline in Tunisia who underwent periodic medical examinations at the Occupational Medicine and Pathology Department in a University Hospital in Sousse. Data collection included socio-demographic information, lifestyle habits, and professional details, gathered through a structured questionnaire. The Sleep Condition Indicator (SCI) was used to screen for insomnia disorder.

**Results:**

The study included 160 participants, predominantly female (60%). Sixty-five percent of the population was over 40 years old. A significant majority (58.8%) were smokers, while 41.3% consumed alcohol. Coffee consumption was high, with 84.4% of participants reporting regular consumption. Regarding professional data, 71.3% of participants were flight attendants, with a median length of service of 15 years. The majority of participants (65%) had flown medium-haul flights (less than 5 hours) in the preceding month. Insomnia was reported in 23.1% of participants.

**Conclusions:**

Our findings highlight the importance of screening for sleep disorders through periodic medical examinations which can significantly contribute to improving sleep quality, enhancing alertness, and ultimately enhancing aviation safety.

**Disclosure of Interest:**

None Declared

